# Combination of lysine‐specific demethylase 6A (KDM6A) and mismatch repair (MMR) status is a potential prognostic factor in colorectal cancer

**DOI:** 10.1002/cam4.3602

**Published:** 2020-11-11

**Authors:** Xiaying Chen, Zuyi Yang, Jiao Feng, Ting Duan, Ting Pan, Lili Yan, Ting Jin, Yu Xiang, Mingming Zhang, Peng Chen, Wengang Wang, Ruonan Zhang, Bi Chen, Liping Zhao, Tian Xie, Xinbing Sui

**Affiliations:** ^1^ College of Pharmacy and Department of Medical Oncology the Affiliated Hospital of Hangzhou Normal University School of Medicine Hangzhou Normal University Hangzhou Zhejiang China; ^2^ Key Laboratory of Elemene Class Anti‐Cancer Chinese Medicines Engineering Laboratory of Development and Application of Traditional Chinese Medicines Collaborative Innovation Center of Traditional Chinese Medicines of Zhejiang Province Hangzhou Normal University Hangzhou Zhejiang China

## Abstract

**Purpose:**

To evaluate the relationship between the DNA mismatch repair (MMR) status and histone lysine‐specific demethylase 6A (KDM6A) on the prognosis of colorectal cancer (CRC).

**Methods:**

About 594 patients with CRC from The Cancer Genome Atlas (TCGA) were enrolled in this retrospective study. Subsequently, a series of different classification tests for MMR status, cancer types, and target gene expression was conducted.

**Results:**

After filtering out the KDMs group of genes, we selected KDM6A as the target gene. A significant difference in the performance of KDM6A in tumor and normal tissues were confirmed. Our results showed a lower KDM6A expression, lower KDM6A exon expression, and higher KDM6A DNA methylation than their corresponding normal tissues in colon adenocarcinoma (COAD). Notably, the main MMR genes were highly expressed in tumor tissues than normal tissues both in COAD and rectum adenocarcinoma (READ). Moreover, proficient DNA mismatch repair (pMMR) was found to be an important poor prognostic factor in COAD (*p* = 0.0064) and the low KDM6A expression was an important factor for poor prognosis in READ (*p* = 0.0217). Based on these results, we consequently relate MMR status with KDM6A expression in predicting the prognosis of patients with CRC. Moreover, patients with pMMR exhibited a low KDM6A expression in COAD (*p* = 0.0250). Samples were divided into two groups based on the KDM6A expression. Interestingly, the group with low KDM6A expression showed no difference between pMMR and deficient DNA mismatch repair (dMMR) in prognosis, whereas the group with high KDM6A expression was closely related to MMR status in OS (*p* = 0.0082). Besides, COAD patients with high KDM6A expression and pMMR status had poor OS (*p* = 0.0082).

**Conclusions:**

The KDM6A/MMR classification‐based subtypes of low KDM6A expression/READ, high KDM6A expression/pMMR, and COAD/pMMR were associated with poor prognosis. This classification can be a novel prognostic approach in CRC.

## Graphical abstract

The KDM6A/MMR classification based subtypes of low KDM6A expression/READ, high KDM6A expression/pMMR, and COAD/pMMR were associated with poor prognosis. This classification could more accurately stratify CRC patients as per the prognostic performance.

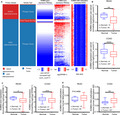

## 1 INTRODUCTION

Globally, colorectal cancer (CRC) is among the most common malignancy,^1^ ranked third in terms of incidence but second in terms of mortality.^2^ Approximately 1.2 million people are annually diagnosed with CRC worldwide, however, over 600,000 patients directly or indirectly succumbed to CRC.^3^ Several studies have discovered risk markers related to CRC prognoses, such as TNM staging, age, surgical resection range, postoperative radiotherapy, adjuvant chemotherapy, microsatellite instability (MSI), and BRAF mutation.^4^ The DNA mismatch repair (MMR) system which includes four key proteins—mutL homolog 1 (MLH1), mutS homolog 2 (MSH2), mutS homolog 6 (MSH6), and postmeiotic segregation increased 2 (PMS2)—plays an important role in retaining genomic stability. Currently, numerous retrospective studies have confirmed that CRC patients with defective MMR (dMMR) have improved from the stage‐independent survival relative to patients with proficient mismatch repair (pMMR).^5^

Histone lysine demethylases (KDMs) and methyltransferases (KMTs) coordinatively regulate the methylation of lysine residues within histones to maintain cell fate and genomic stability, which underpin gene regulation and several cellular processes.^6^, ^7^ KDM6A (Lysine‐specific Demethylase 6A) and the other KDM subfamily members play vital roles in the development and differentiation, both in vitro and in vivo.^8^, ^9^, ^10^ However, numerous studies have identified the relationship between cancer prognoses and KDM6A mutation or abnormal expression, for instance, in myeloma,^11^ acute lymphoblastic leukemia,^12^ breast cancer,^13^ HPV (human papillomavirus)‐positive tumors,^14^ and esophageal squamous cell carcinoma.^15^, ^16^

Key genetic changes are one of the most important risk factors, not only in CRC, that cause other functional proteins or genes abnormalities such as BRAF,^17^, ^18^, ^19^, ^20^ KRAS,^21^ and TP53. Of concern, there are sufficient valuable molecular markers that potentially predict CRC prognosis as well as distinguish between colon and rectal cancer. Therefore, it is important to identify more diagnostic and prognostic biomarkers to stratify patients into different risk categories, which will allow the advancements and adoption of more specific treatment agents.

To our knowledge, this is the first report that combination of KDM6A and MMR status is a potential prognostic factor in colorectal cancer. This present study demonstrates that KDM6A and MMR can be used as tumor biomarkers for CRC prognosis to analyze different grouped samples from The Cancer Genome Atlas (TCGA).

## 2 MATERIALS AND METHODS

### 2.1 Primary filtering of related genes using GEPIA and UCSC

We examined 24 genes belonging to the KDM family using the Kaplan–Meier survival curves of gene expression by GEPIA (http://gepia.cance​r-pku.cn/index.html)^22^ in CRC. The filtered genes tested in TCGA Colon and Rectal Cancer (COADREAD) using the UCSC Xena browser (https://xenab​rowser.net/)^23^ showed that the presence of KDM6A was significantly different between the tumor and normal tissues. Besides, the genes expression (FPKM) were downloaded from UCSC (https://gdc.xenah​ubs.net/downl​oad/TCGA-COAD.htseq_fpkm.tsv.gz; Full metadata, https://gdc.xenah​ubs.net/downl​oad/TCGA-READ.htseq_fpkm.tsv.gz; Full metadata). The phenotypes of COAD and READ were also downloaded from UCSC. Furthermore, we collected the survival data and determined the loss expression of MMR proteins by immunohistochemistry (IHC) to supplement the information obtained from cBioPortal (https://gdc.xenah​ubs.net/downl​oad/TCGA-COAD.GDC_pheno​type.tsv.gz; Full metadata, https://gdc.xenah​ubs.net/downl​oad/TCGA-READ.GDC_pheno​type.tsv.gz; Full metadata).

### 2.2 Overall survival analysis using GraphPad Prism v7.0

We downloaded multiple data of 594 samples was downloaded in TCGA, including the alteration, copy number, and clinical data (http://www.cbiop​ortal.org/).^24^ Simultaneously, the analysis of KDM6A mutation, percentage of copy number alterations, and mRNA expression Z scores were calculated using GraphPad Prism v7.0.

### 2.3 DNA methylation and exon expression analysis using UCSC

The UCSC Xena browser was used for ease of visualizing the KDM6A mRNA expression, DNA methylation, exon expression, and clinical data, as well as determining the relationships between them with TCGA. Additionally, Welch's t‐test and Kaplan–Meier survival curves were used to analyze the UCSC TCGA Colon and Rectal Cancer data.

## 3 RESULTS

### 3.1 CRC tissues were characterized by lower expression and higher DNA methylation

Based on our results, some DNA methylation proteins were abnormally expressed in many cancers, which affected cancer prognosis. Therefore, we further assessed the KDM protein family with GEPIA (especially, COAD and READ) and obtained the Kaplan–Meier survival curves of gene expression. After checking 24 genes, we found that the expression of KDM6A and KDM7A may be related to OS in CRC. Although the p‐value of OS for both KDM6A and KDM7A were higher than 0.05 (KDM6A: *p* = 0.054, KDM7A: *p* = 0.052), considering the different sample size in the TCGA database owing to a delayed update, the two genes were closely related to OS in CRC (Figure S1). KDM6A and KDM7A expression had difference in OS of COAD and READ (Figure S2). To further identify the selected target gene, we compared the expression of KDM‐coding genes between the tumor and normal tissues in TCGA Colon and Rectal Cancer using UCSC. The results showed that only KDM6A, KDM6B, KDM4A, and KDM4B were downregulated in CRC tissues (Figure S3). Therefore, we selected KDM6A as the target gene.

**Figure 1:**
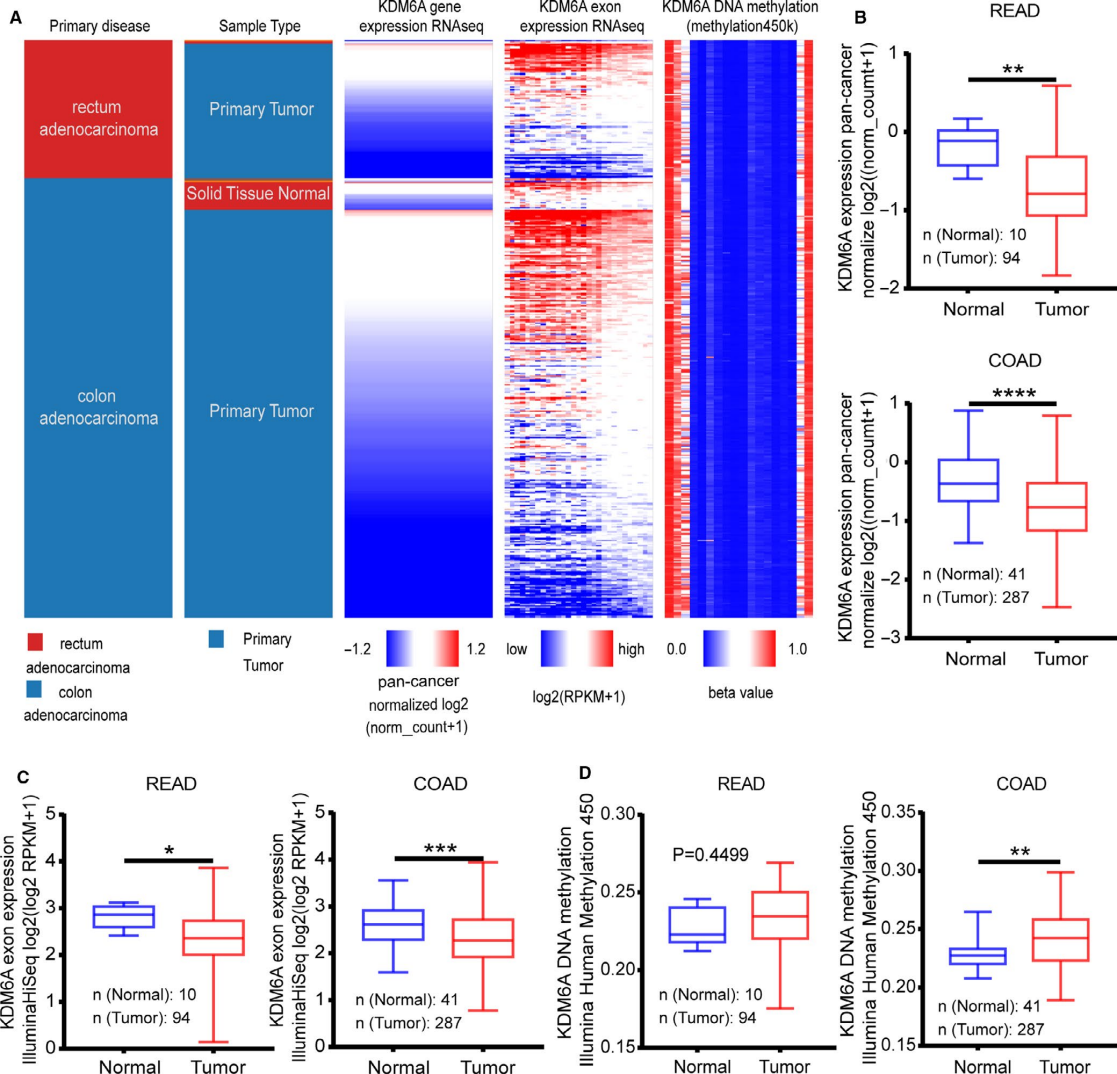
Colon adenocarcinoma (COAD) tissues showing a lower KDM6A expression and higher DNA methylation than the corresponding normal tissues. (A) Heatmap analysis used to compare gene expression, exon expression, and DNA methylation of KDM6A by UCSC. (B) Unpaired*t*‐test showing downregulation of KDM6A in tumor tissues was less in READ (*p* = 0.0017) than in COAD (*p* < 0.0001). (C) Downregulation of KDM6A exon expression in tumor tissues was commonly found in READ (*p* < 0.0001) and COAD (*p* < 0.0001) (D) A higher KDM6A DNA methylation in tumor tissues in COAD (*p* = 0.0014) tissues and not in READ (*p* = 0.4499) tissues

To determine the differences in KDM6A expression between tumor and normal tissues, the heatmap was generated from UCSC to compare their gene expression, exon expression, and DNA methylation (Figure 1A). Boxplot analysis between tumor and normal tissues showed a lower KDM6A expression (Figure 1B), lower KDM6A exon expression (Figure 1C), and higher KDM6A DNA methylation (Figure 1D) in COAD (*p* < 0.0001, *p* = 0.0009, *p* = 0.0014), and READ (*p* = 0.0017, *p* = 0.0203, *p* = 0.4499).

### 3.2 Major MMR genes showed higher expression in CRC tissues compared with normal tissues

**Figure 2:**
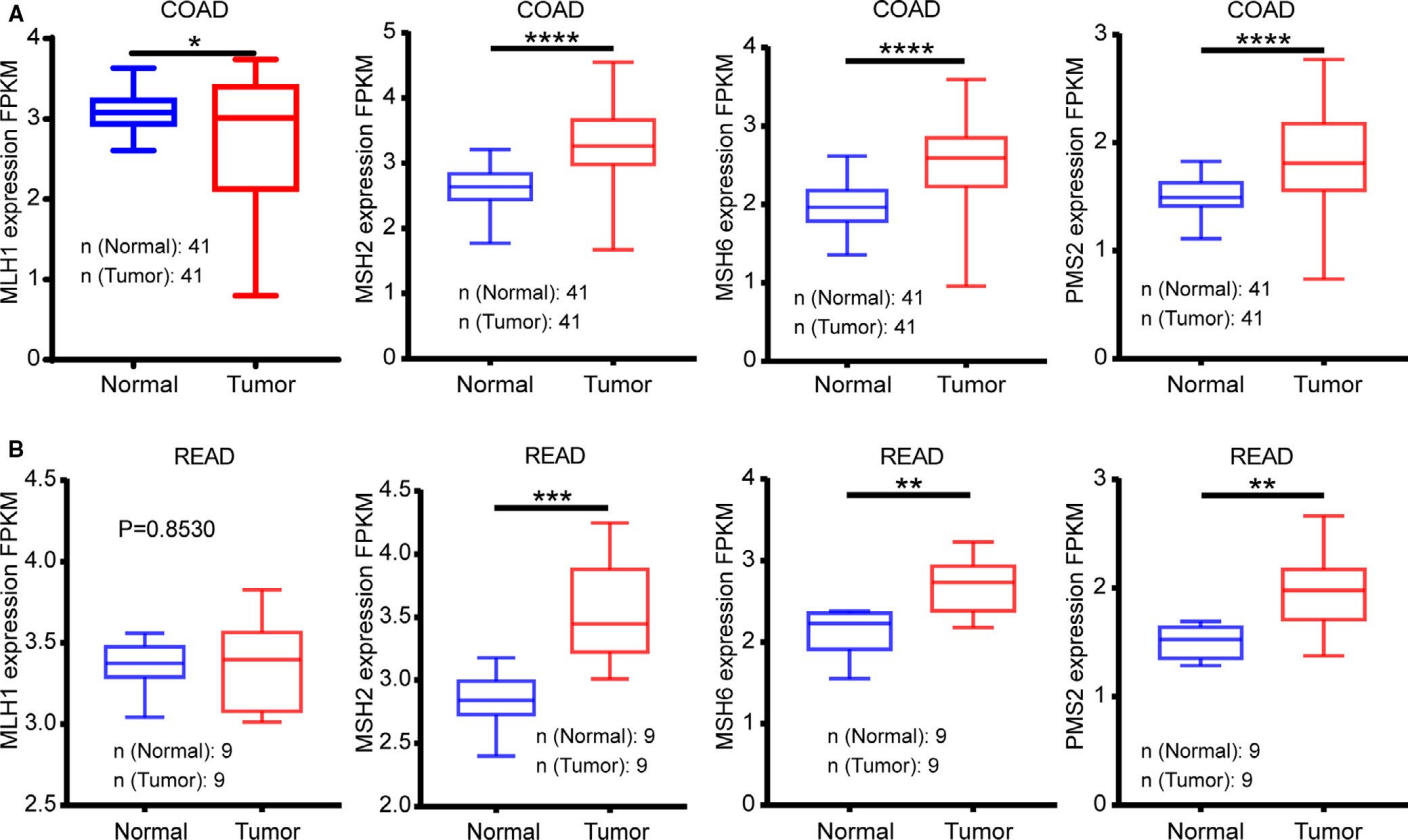
Expression trends of main mismatch repair (MMR) genes compared in tumor vs. normal tissues. (A) Tumor tissues with an overexpressed MSH2, MSH6, and PSM2 (*p* < 0.0001) and an under‐expressed MLH1 (*p* = 0.0142) in colon adenocarcinoma (COAD) as compared with normal tissues. (B) MSH2 (*p* = 0.0007), MSH6 (*p* = 0.0042) were apparent overexpressed in READ tissues, whereas PSM2 (*p* = 0.0060) had no significant difference (*p* = 0.8530)

MLH1, MSH2, MSH6, and PSM2 are the main functional genes that mediate key roles in the MMR mechanism. Following the comparative analysis of the expression of these genes between tumor and paired normal tissues in TCGA, MSH2, MSH6, and PSM2 were markedly overexpressed (*p* < 0.0001), whereas MLH1 was significantly downregulated (*p* = 0.0142, Figure 2A) in COAD. Therefore, the MMR status seemed to be of immense influence in COAD patients. Similarly, MSH2 (*p* = 0.0007), MSH6 (*p* = 0.0042), and PSM2 (*p* = 0.0060) were overexpressed but MLH1 showed no difference (*p* = 0.8530, Figure 2B) in READ samples. From the expression of the MMR genes using IHC, over one‐third of the patients with COAD showed pMMR status (16/41). Interestingly, only two READ patients (2/9) had pMMR status.

### 3.3 Low expression of KDM6A was an important prognostic factor for poor OS in READ

**Figure 3:**
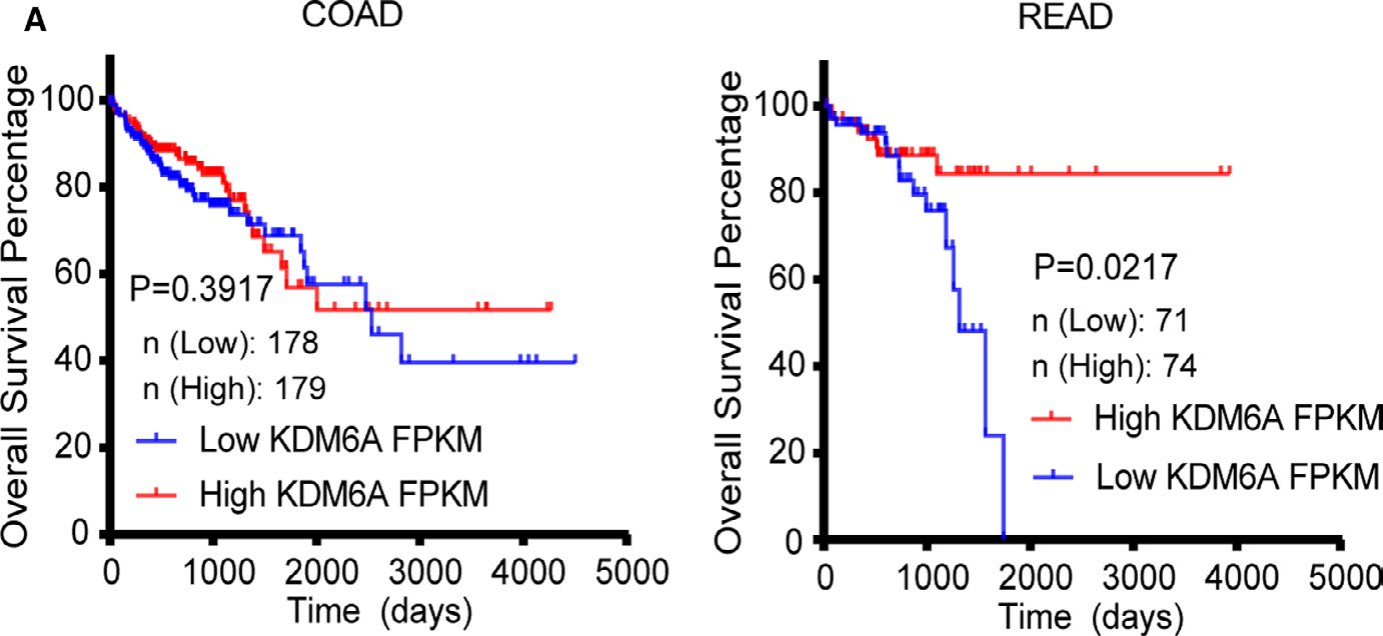
Gene expression and prognostic relevance of KDM6A in the TCGA cohort of CRC. (A) Kaplan–Meier survival analysis showing KDM6A expression was significantly correlated with OS in READ and low KDM6A expression was a poor prognostic factor, but not in colon adenocarcinoma (COAD)

Given consistency and ease of accessing various data, we downloaded TCGA pan‐cancer data from cBioPortal. Although the result analyzed by GEPIA indicated no significant differences between KDM6A expression and OS, we still used our data set to reanalysis. Moreover, results from the survival curves revealed that CRC patients with low KDM6A expression had a significantly worse OS (*p* = 0.0279) than those with low KDM6A expression. Hence, we further assessed the potential of KDM6A expression as an important factor in COAD and READ. Consequently, our results showed that low KDM6A expression was a prognostic factor for poor OS in READ (*p* = 0.0217; low KDM6A expression/high KDM6A expression: 71/74) but not in COAD (low KDM6A expression/high KDM6A expression: 178/179; Figure 3A). Next, we tried to investigate the underlying mechanisms of dysregulated KDM6A expression in READ and COAD.

### 3.4 pMMR was an important prognostic factor for poor OS

**Figure 4:**
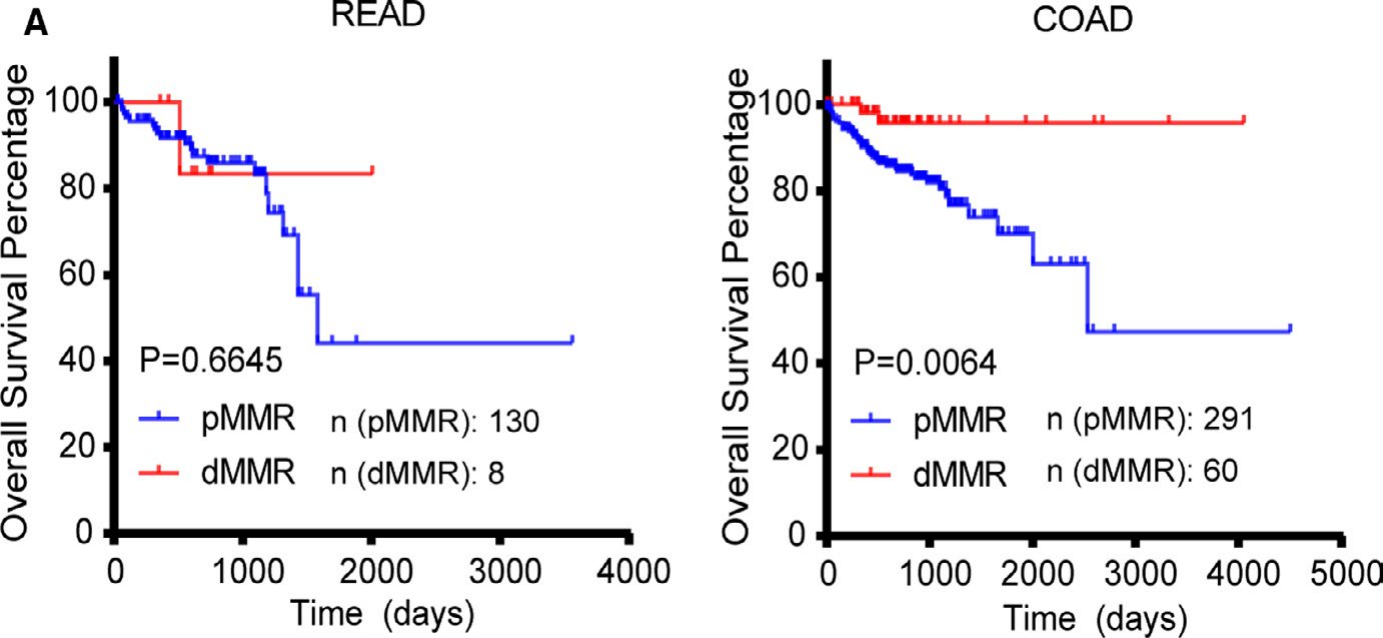
Mismatch repair (MMR) status and prognostic relevance of CRC. (A) Kaplan–Meier survival analysis showed that pMMR was significantly correlated with poor OS in colon adenocarcinoma (COAD), but not in READ

Herein, we explored the association between MMR status and survival in READ and COAD patients. With the same data set, we found that patients with pMMR were predicted to have a poorer prognosis in CRC than those with dMMR (*p* = 0.0095), which was likely a result of immune resistance in pMMR patients.^25^ In evaluating the different prognosis of MMR status in READ and COAD, the OS time for patients was assessed, whereby we generated the Kaplan–Meier survival curves using GraphPad Prism v7.0. Our results showed that that pMMR status was significantly different in COAD (*p* = 0.0064; dMMR/pMMR: 60/291), but not in READ (*p* = 0.6645; dMMR/pMMR: 8/130; Figure 4A).

### 3.5 Relationship between high expression of KDM6A and MMR proteins in COAD prognosis

**Figure 5:**
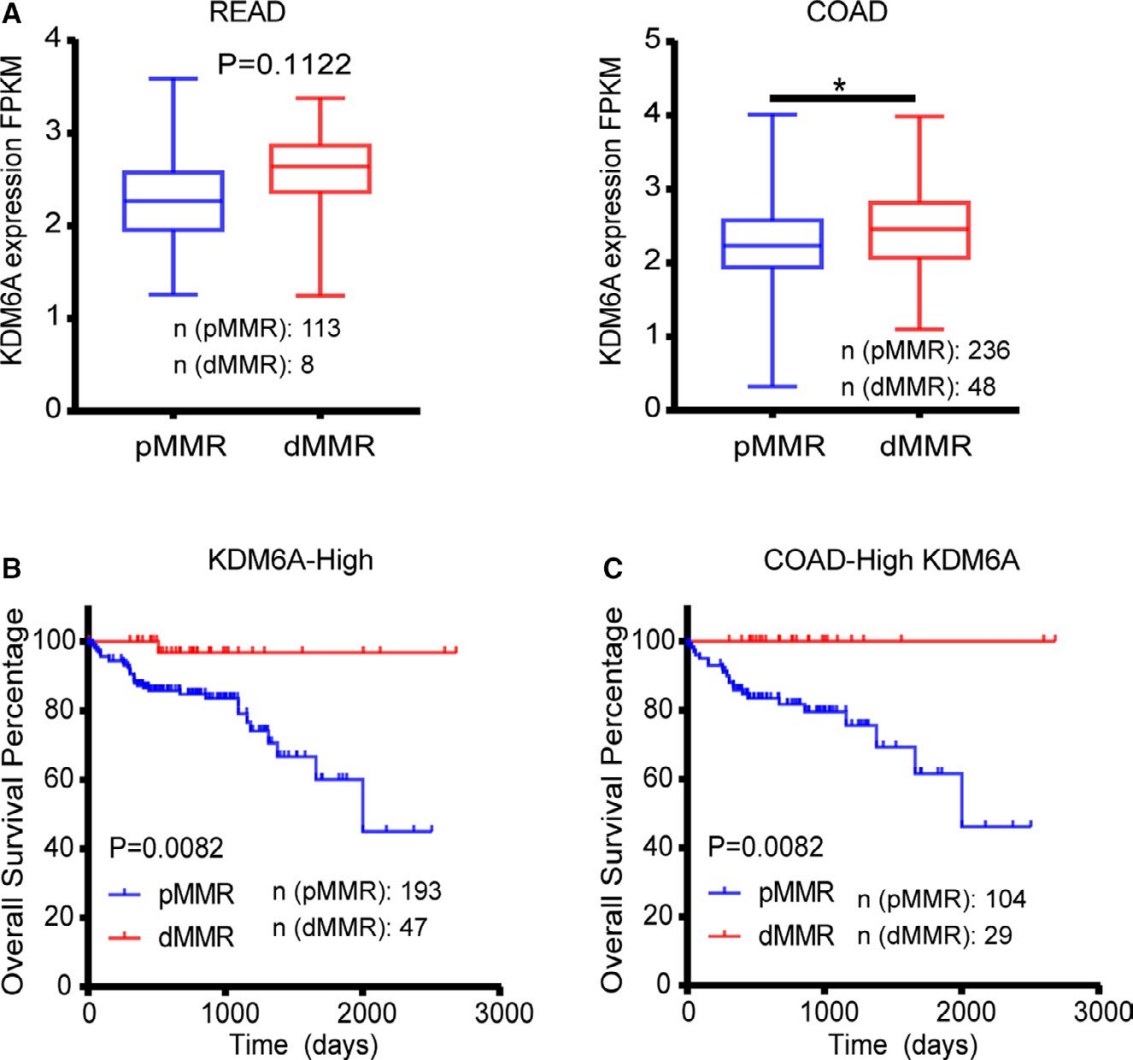
Mismatch repair (MMR) status is likely related to KDM6A expression. (A) colon adenocarcinoma (COAD) patients with pMMR status had lower KDM6A expression than those with dMMR status. (B) In the high KDM6A expression group, MMR status was significantly related to OS. (C) In the COAD patients with low KDM6A expression, pMMR predicted poorer OS

KDM6A showed lower expression in tumor tissues than in normal tissues in COAD, and pMMR was a poor prognostic marker in COAD. Therefore, we hypothesized that KDM6A expression and MMR status are related in COAD. Thus, we first confirmed that CRC patients with pMMR had a lower KDM6A expression (*p* = 0.002). The COAD patients with pMMR status were significantly different to those with dMMR (*p* = 0.025; pMMR/dMMR: 236/48) but none in READ (*p* = 0.1122; pMMR/dMMR: 113/8; Figure 5A). We divided samples (N = 590) into two groups according to KDM6A expression. In the high KDM6A expression group (N = 240), the pMMR had a worse OS than dMMR (*p* = 0.0086; pMMR/dMMR: 193/47; Figure 5B). However, in the low KDM6A expression group (N = 239), the pMMR was not significantly different from that of dMMR in prognosis (*p* = 0.4906’ pMMR/dMMR: 218/21). Furthermore, we explored the detailed influence of cancer type in this pair of factors. From our results, we determined that patients with high KDM6A expression in COAD showed more relevance to MMR status (*p* = 0.0082; pMMR/dMMR: 104/29) than those with low KDM6A expression (*p* = 0.5401; pMMR/dMMR: 120/18; Figure 5C). On the one hand, there was no significant difference in prognosis between pMMR and dMMR in READ patients with high KDM6A expression (*p* = 0.8049; pMMR/dMMR: 52/7). Besides, we could not obtain sufficient data from READ patients with dMMR (only 1/78 samples). Therefore, our results were inconclusive to ascertain the relationship of MMR status to prognosis in this group.

## 4 DISCUSSION

Colorectal cancer continues to have high morbidity and mortality, there is about threefold higher in transitioned versus transitioning countries in incidence rates in several areas (2). So far, there are extensive studies that have assessed methods for the diagnosis and treatment. MMR status, one of the prognostic markers for CRC, is an important tumor suppressor pathway that is lost in 10–40% of sporadic cancers.^26^, ^27^, ^28^, ^29^ Analysis of MMR proteins expression by IHC is a particular method to identify suitable drugs for CRC patients. As we know, dMMR patients cannot benefit from fluorouracil and immunosuppressive drugs such as pembrolizumab, nivolumab, and other target drugs. pMMR was revealed as a poor prognostic factor in CRC,^5^ which was consistent with our present results. The present study demonstrated that pMMR was a poor prognostic factor only in COAD. However, dMMR proteins are observed only in about 13% CRC patients,^26^, ^27^, ^28^, ^29^, ^30^ which shows that it cannot be a universal marker for the entire spectrum of CRC.

Therefore, the development of new diagnostic and prognostic biomarkers for managing patients with CRC is urgently needed.

The KDM family genes, which have been extensively explored in recent years, may harbor candidate genes as prognostic biomarkers. Thus, we filtered 24 KDM‐coding genes by UCSC and GEPIA. In the primary filtering using TCGA data, we found that the mRNA levels of KDM6 (KDM6A and KDM6B) and KDM4 (KDM4A and KDM4B) were significantly downregulated in CRC tissues as compared to their adjacent normal tissues. Since the expression of KDM7A and KDM6A was potentially related to OS in CRC, we considered KDM6A as the target gene. There were more significant differences in KDM6A mRNA expression, exon expression, and DNA methylation in CRC tissue (especially in COAD) compared to normal tissues. However, low KDM6A expression was an important prognostic factor for poor OS in READ but not in COAD. This suggested that the MMR status would be combined to fill the gap in prognosis.

Simultaneously, KDM6A expression in COAD tissues was markedly lower, and pMMR was related to the prognosis of COAD. Hence, we evaluated the KDM6A expression of different MMR status in tumor tissues. In our study, pMMR was associated with lower KDM6A expression in COAD and predicted worse prognosis in CRC patients with higher KDM6A expression. Interestingly, COAD patients with high KDM6A expression showed more relevance to MMR status than those with low KDM6A expression. As for READ, the sample size was extremely small for reliable results. Therefore, findings from the present work suggested that high KDM6A expression and pMMR may be associated with COAD.

Collectively, the above findings demonstrated that the downregulation of KDM6A expression was associated with the pathogenesis of rectal cancer, whereas the high KDM6A expression combined with MMR status could be utilized as a potential biomarker for CRC prognosis.

## CONFLICT OF INTEREST

The authors declare that the research was conducted in the absence of any commercial or financial relationships that could be construed as a potential conflict of interest.

## AUTHOR CONTRIBUTIONS

X.S. and T.X. guided and designed the research; X.C. performed most of research. Z.Y., J.F., and T.D. provided major technical supports. T.P., L.Y., T.J., Y.X., and W.W. contributed materials information gathering and data analysis. L.Z., M.Z., P.C., R.Z., and B.C. collected and sorted the data. X.S. and X.C. wrote manuscript with contributions from the other authors.

## Supporting information

Fig S1

Fig S2

Fig S3
