# Supplementary figures and images for: Combination of lysine‐specific demethylase 6A (KDM6A) and mismatch repair (MMR) status is a potential prognostic factor in colorectal cancer
Source: Cancer Med. 2020 Nov 11;10(1):317–24. doi: 10.1002/cam4.3602 (PMC7826484; doi:10.1002/cam4.3602)

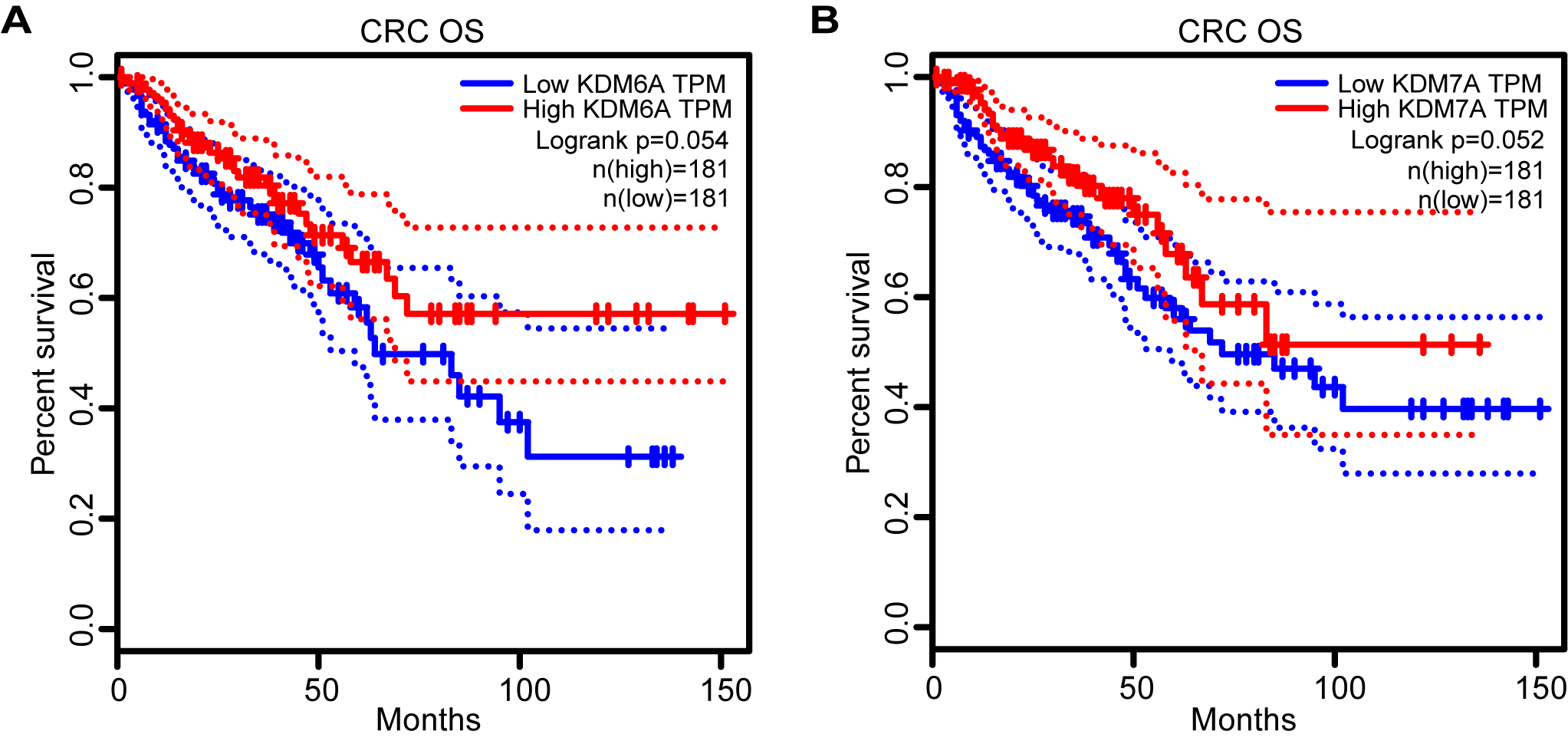

Supplement: Supplementary file 1 — Fig S1 [file CAM4-10-317-s001.tif]

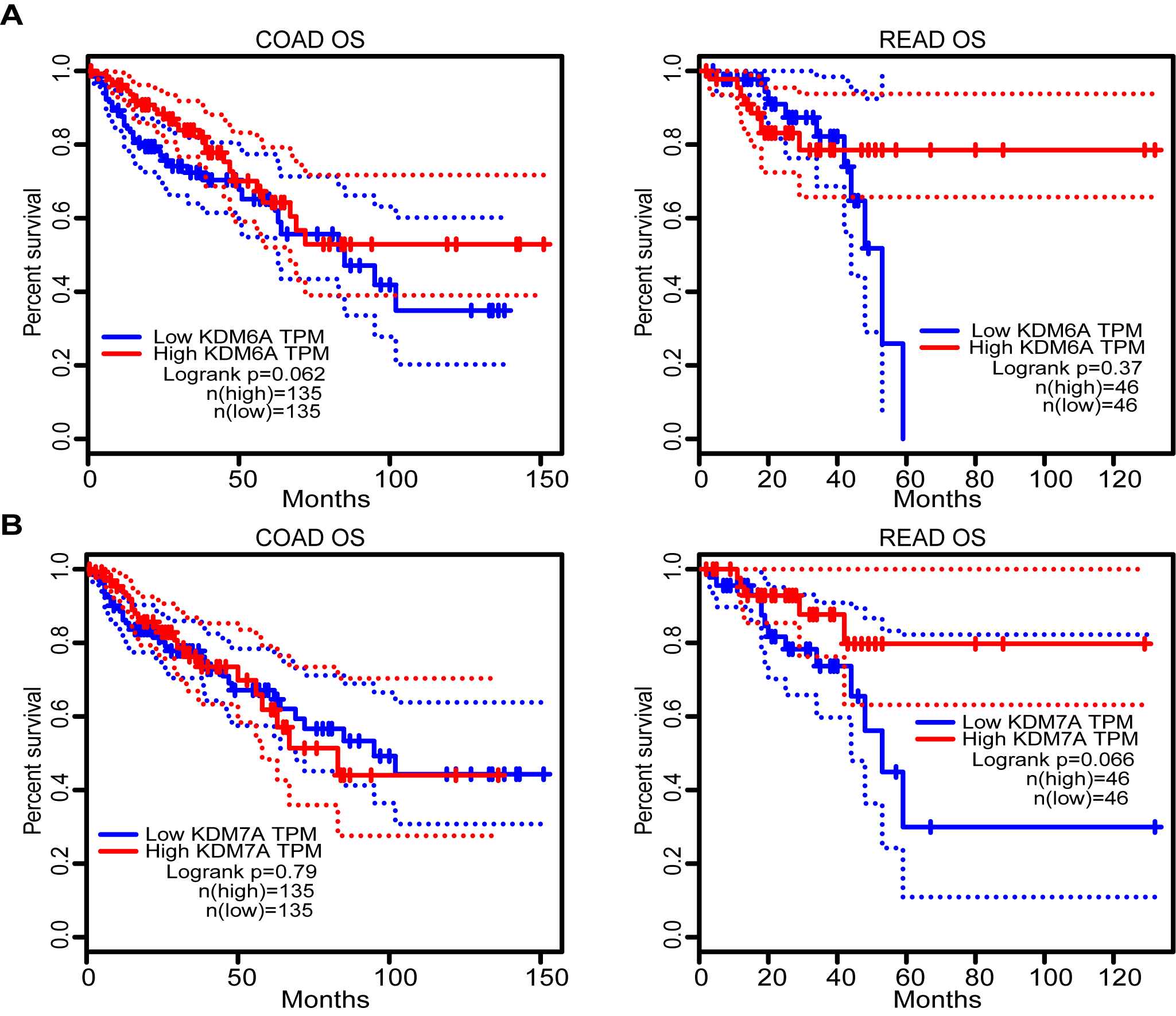

Supplement: Supplementary file 2 — Fig S2 [file CAM4-10-317-s002.tif]

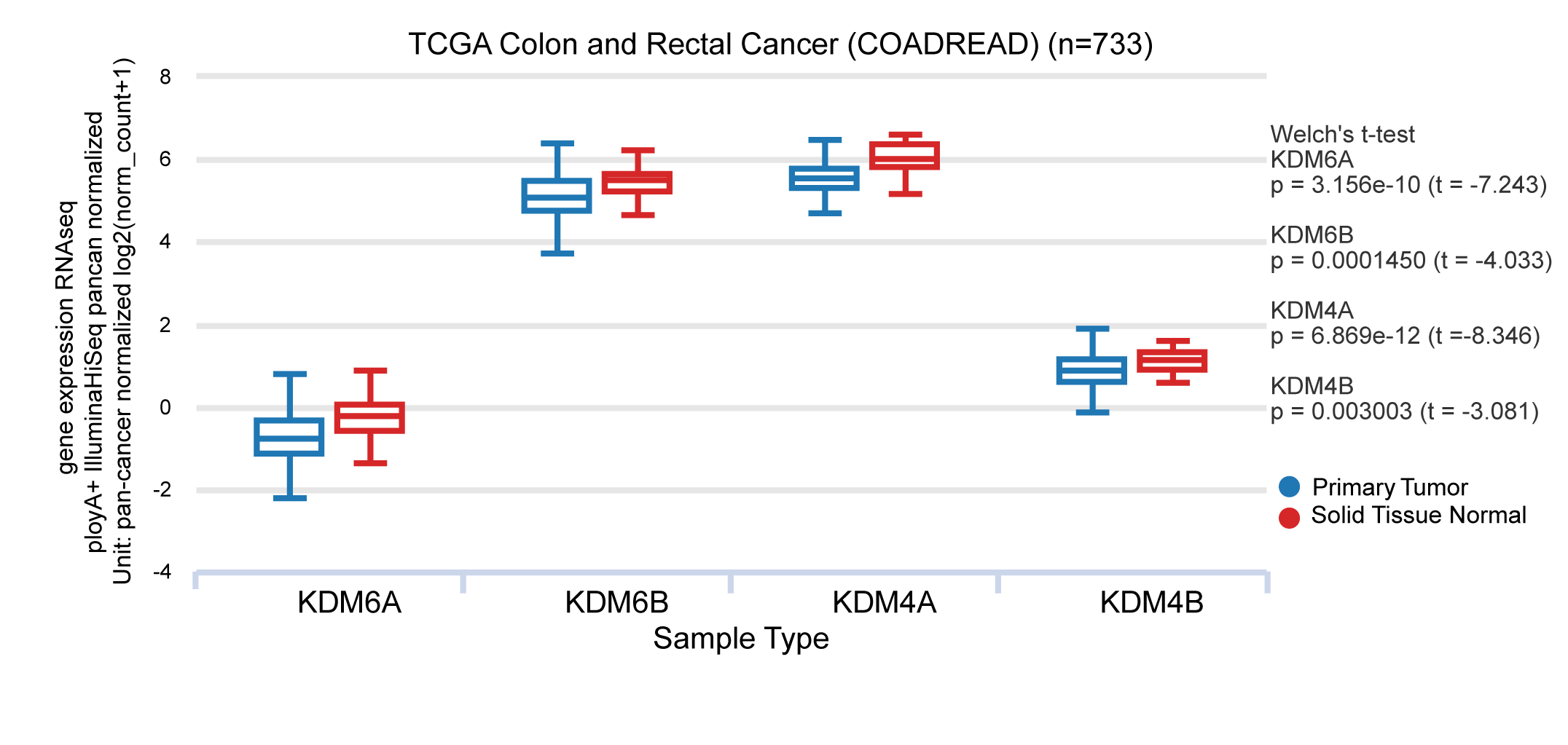

Supplement: Supplementary file 3 — Fig S3 [file CAM4-10-317-s003.tif]
